# Effusion‐Dominant Pulmonary Sarcomatoid Carcinoma Without a Primary Mass: A Case Report

**DOI:** 10.1002/rcr2.70459

**Published:** 2026-01-05

**Authors:** Sze Kye Teoh, Yen Shen Wong, Yiet Fai Ng, Yu Wei Cheah, Suhashini Ganapaty, Saiful Safuan Md Sani

**Affiliations:** ^1^ Respiratory Unit, Department of Internal Medicine Ampang Hospital Pandan Indah Selangor Malaysia; ^2^ Faculty Of Medicine University Teknologi MARA (UiTM) Sungai Buloh Selangor Malaysia; ^3^ Department Of Pathology Hospital Sultan Idris Shah Serdang Selangor Malaysia

**Keywords:** immunohistochemistry, malignant pleural effusion, Sarcomatoid carcinoma, thoracoscopy

## Abstract

Sarcomatoid carcinoma is a rare, aggressive lung cancer subtype. It typically presents as a pulmonary or pleural mass. Effusion‐dominant disease without an identifiable primary mass is exceptionally uncommon. We report a 59‐year‐old man presenting with dyspnoea, chest pain, and fever. Imaging showed right pleural effusion without a lung mass. Thoracoscopy revealed necrotic pleural nodules. Histology demonstrated spindle‐shaped atypical cells, positive for cytokeratin AE1/AE3 and weakly positive for TTF‐1, but negative for mesothelial markers, consistent with sarcomatoid carcinoma of probable pulmonary origin. Staging CT revealed contralateral lung nodules, mediastinal lymphadenopathy, and distant metastases, but no dominant primary lesion. The patient declined systemic therapy and received palliative care. Sarcomatoid carcinoma presenting as malignant pleural effusion without a mass is rare. Early thoracoscopy and histological confirmation are crucial. Prognosis remains poor, though emerging data suggest a role for immunotherapy.

## Graphical abstract

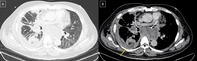

## 1 Introduction

Pulmonary sarcomatoid carcinoma (PSC) accounts for only 0.1%–1% of non‐small cell lung cancers (NSCLC) [1, 2]. It is characterised by spindle or giant cell morphology and demonstrates both epithelial and mesenchymal features [1, 2]. PSC is associated with aggressive behaviour and poor prognosis, with median survival typically less than one year [2, 3]. Most patients present with a pulmonary or pleural mass, often accompanied by pleural effusion [2]. In contrast, effusion‐dominant disease without an identifiable primary lesion is extremely rare and diagnostically challenging [4, 5].

We describe a patient with high‐grade sarcomatoid carcinoma presenting as malignant pleural effusion without a primary mass. This case highlights the diagnostic challenges, aggressive clinical course, and importance of early biopsy. We also review the limited literature describing similar presentations.

## 2 Case Report

**FIGURE 1:**
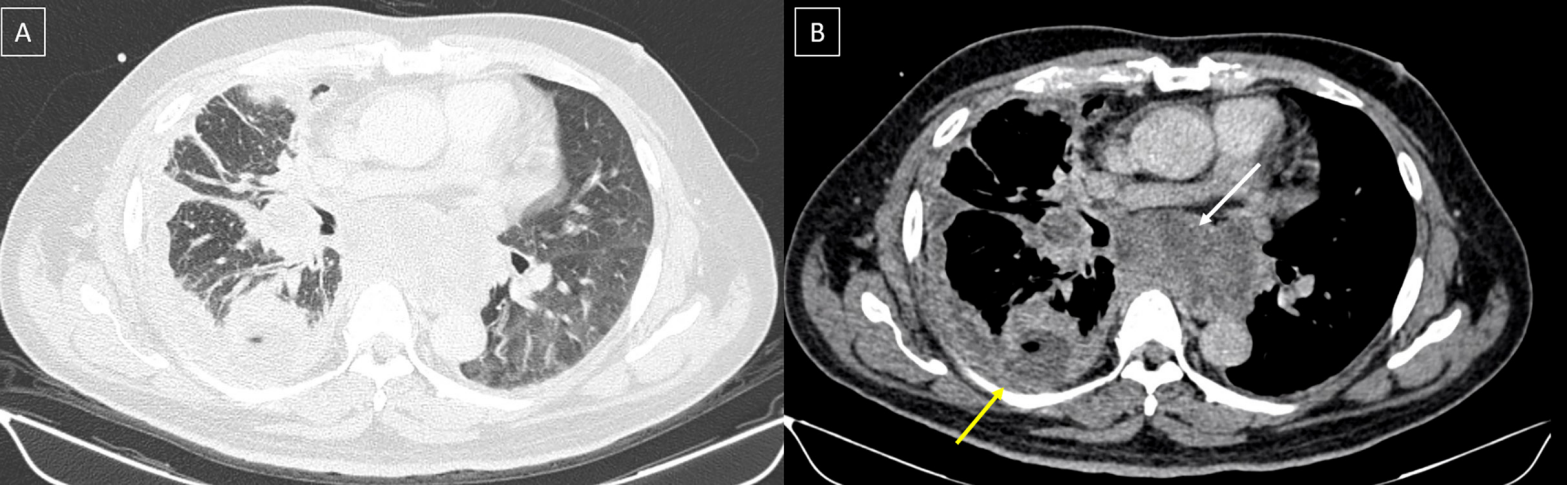
Imaging findings of the patient. Axial contrast‐enhanced CT of the thorax: Lung window (A) and soft‐tissue window (B) demonstrating a loculated right pleural effusion (yellow arrow) and mediastinal lymphadenopathy (white arrow).

A 59‐year‐old Malaysian man, an ex‐smoker with a history of hypertension, dyslipidaemia, and obstructive sleep apnoea, presented with a four‐day history of dry cough, fever, progressive dyspnoea, and right‐sided chest pain. He worked as a taxi driver with no known occupational exposure to established lung carcinogens such as asbestos, silica, or heavy metals. Chest radiography demonstrated a large right pleural effusion. Thoracentesis yielded exudative fluid with markedly elevated pleural fluid protein (55 g/L), lactate dehydrogenase (1113 U/L), and lactate (4.8 mmol/L). Microbiological studies, including tuberculosis polymerase chain reaction (TB‐PCR), were negative. Bedside thoracic ultrasound demonstrated multiseptated effusion with irregular pleural thickening and discrete pleural nodules. Contrast‐enhanced CT of the thorax, abdomen, and pelvis revealed diffuse right pleural thickening, contralateral pulmonary nodules, mediastinal lymphadenopathy, and distant metastases involving the liver, peritoneum, adrenal gland, and skeletal muscle (Figure 1). Although no dominant pulmonary mass was identified, both CT and sonographic features are recognised markers of malignant pleural effusion and guided the decision to proceed directly to thoracoscopy for tissue diagnosis.

**FIGURE 2:**
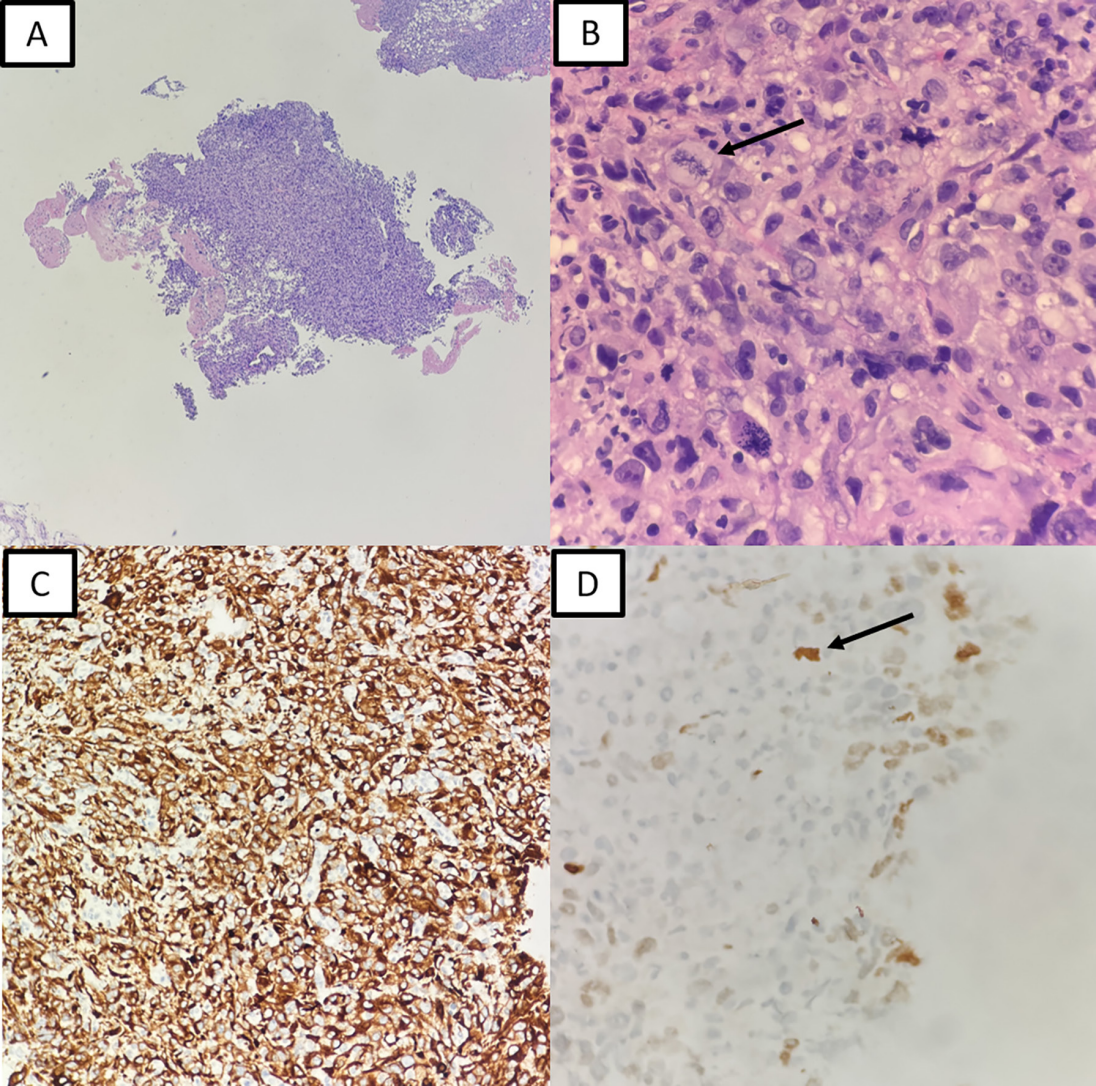
Histological findings from right thoracoscopy. (A) Cellular fragments composed of pleomorphic spindle‐shaped cells. (B) High‐grade nuclear atypia with atypical mitoses (black arrow). (C) Immunohistochemistry demonstrating diffuse positivity for cytokeratin AE1/AE3. (D) Focal positivity for TTF‐1 (black arrow).

Medical thoracoscopy revealed multiseptated pleural cavities with necrotic nodules. Histological examination of pleural biopsies demonstrated spindle to plump atypical cells with hyperchromatic nuclei and frequent mitoses. Immunohistochemistry showed diffuse positivity for cytokeratin AE1/AE3 and focal positivity for TTF‐1, while mesothelial markers (calretinin, WT1) and adenocarcinoma markers (Napsin A, Ber‐EP4) were negative (Figure 2). These findings were consistent with sarcomatoid carcinoma of probable pulmonary origin. No giant cell component, heterologous sarcomatous elements, or biphasic epithelial–mesenchymal architecture was identified, and therefore the tumor was categorized as sarcomatoid carcinoma, spindle‐cell predominant, not otherwise classifiable according to WHO 2021 criteria.

The patient declined systemic chemotherapy and was managed with best supportive care, deteriorating rapidly and succumbing to the disease within weeks.

## 3 Discussion

Pulmonary sarcomatoid carcinoma (PSC) is a rare and aggressive subtype of NSCLC [1, 2], representing only 0.1%–1% of cases. Histologically, epithelial differentiation (cytokeratin positivity) helps distinguish it from true sarcomas, while negative mesothelial markers aid in excluding sarcomatoid mesothelioma [2, 6]. In our patient, the immunoprofile (CK AE1/AE3+, weak TTF‐1+) supported a pulmonary origin [6]. Comprehensive molecular profiling is increasingly recognized as an essential component of evaluating pulmonary sarcomatoid carcinoma, as actionable alterations such as MET exon 14 skipping, ROS1 rearrangements, KRAS mutations, and high PD‐L1 expression may guide targeted therapy or immunotherapy. However, real‐world barriers including limited tissue availability, delayed presentation, rapid clinical decline, and financial constraints often prevent full genomic workup in patients with aggressive disease. Our patient deteriorated soon after diagnosis and opted not to proceed with additional paid testing, illustrating a common challenge in resource‐limited settings. This underscores the importance of early tissue acquisition, adequate sampling, and timely molecular analysis when sarcomatoid carcinoma is suspected.

Effusion‐dominant PSC without a primary lung mass is exceedingly rare. Only a few cases have been documented, including those reported by Sasaki et al., Chua et al., and Sugimoto et al. [4, 5, 7]. Our case is unique in demonstrating rapid systemic metastases (liver, peritoneum, adrenal, and skeletal muscle) in the absence of a detectable pulmonary mass, suggesting aggressive biology and possible early dissemination before formation of a dominant lesion.

In regions with high tuberculosis prevalence, malignant effusions without an obvious lung mass are often initially attributed to tuberculous pleurisy. Likewise, effusion‐dominant PSC may be mistaken for mesothelioma or metastatic sarcoma. Such misinterpretations can delay diagnosis and appropriate management. This case reinforces the importance of early thoracoscopy and comprehensive immunohistochemistry in unexplained exudative effusions to avoid unnecessary empirical anti‐TB or antibiotic treatment.

PSC is generally resistant to conventional chemotherapy, with limited survival benefit [3, 8]. Recent evidence suggests potential benefit from immune checkpoint inhibitors (ICIs), particularly in patients with high PD‐L1 expression. Moreover, molecular profiling has identified targetable alterations such as *ROS1 fusions, MET exon 14 skipping mutations, and KRAS mutations* [3, 8]. Chua et al. highlighted a ROS1‐rearranged PSC responding to targeted therapy [5]. Although our patient declined systemic treatment, future management of similar cases should consider early molecular testing and immunotherapy referral, even when no primary mass is detected.

In conclusion, PSC presenting as malignant pleural effusion without a dominant mass is exceptionally rare and carries a poor prognosis. Early thoracoscopy with biopsy is essential for accurate diagnosis, particularly in TB‐endemic regions where misdiagnosis is common. Molecular profiling and timely access to immunotherapy should be considered to improve outcomes in this aggressive disease.

## Author Contributions

All listed authors contributed to the article.

## Funding

The authors have nothing to report.

## Consent

The authors declare that written informed consent was obtained for the publication of this manuscript and accompanying images and attest that the form used to obtain consent from the patient complies with the Journal requirements as outlined in the author guidelines.

## Conflicts of Interest

The authors declare no conflicts of interest.

## Data Availability

The data that support the findings of this study are available from the corresponding author upon reasonable request.
